# Localization of the epileptogenic network from scalp EEG using a patient-specific whole-brain model

**DOI:** 10.1162/netn_a_00418

**Published:** 2025-03-03

**Authors:** Mihai Dragos Maliia, Elif Köksal-Ersöz, Adrien Benard, Tristan Calas, Anca Nica, Yves Denoyer, Maxime Yochum, Fabrice Wendling, Pascal Benquet

**Affiliations:** University of Rennes, INSERM, LTSI-U1099, Rennes, France; “Van Gogh” Epilepsy Surgery Unit, Neurology Department, CIC 1414, University Hospital, Rennes, France; Neurology Department, Lorient Hospital, Lorient, France

**Keywords:** Focal cortical dysplasia (FCD), EEG modeling, Digital brain, Epilepsy surgery, ECoG, Interictal epileptiform discharges (IED)

## Abstract

Computational modeling is a key tool for elucidating the neuronal mechanisms underlying epileptic activity. Despite considerable progress, existing models often lack realistic accuracy in representing electrophysiological epileptic activity. In this study, we used a comprehensive human brain model based on a neural mass model, which is tailored to the layered structure of the neocortex and incorporates patient-specific imaging data. This approach allowed the simulation of scalp EEGs in an epileptic patient suffering from type 2 focal cortical dysplasia (FCD). The simulation specifically addressed epileptic activity induced by FCD, faithfully reproducing intracranial interictal epileptiform discharges (IEDs) recorded with electrocorticography. For constructing the patient-specific scalp EEG, we carefully defined a clear delineation of the epileptogenic zone by numerical simulations to ensure fidelity to the topography, polarity, and diffusion characteristics of IEDs. This nuanced approach improves the accuracy of the simulated EEG signal, provides a more accurate representation of epileptic activity, and enhances our understanding of the mechanism behind the epileptogenic networks. The accuracy of the model was confirmed by a postoperative reevaluation with a secondary EEG simulation that was consistent with the lesion’s removal. Ultimately, this personalized approach may prove instrumental in optimizing and tailoring epilepsy treatment strategies.

## Author Summary

This study aimed to create a neurophysiologically grounded computer model of focal epilepsy. This is a feature frequently lacking to simulations in this domain, making the translation from in silico to in vivo results questionable and difficult to understand for clinical electrophysiologists. We adapted a whole-brain neuronal mass model for EEG generation in various conscious states to replicate the EEG patterns of a type 2 focal cortical dysplasia (FCD), a condition associated with epilepsy. Our model successfully simulated both intracranial and scalp EEGs of a complex patient with type 2 FCD, who was later cured through surgery. Importantly, the simulated lesion location matched the patient’s epileptogenic zone, and removing this area in the model eliminated epileptic activity in the EEG, demonstrating the model’s accuracy.

## INTRODUCTION

Computational modeling is a well-established method to provide insights on the neurophysiology behind epileptic activity (Lopes da Silva et al., 2003; Suffczynski et al., 2004; Traub & Wong, 1982; Wendling et al., 2016). Neurophysiologically plausible models allow to propose testable hypotheses about the key role of glutamatergic and GABAergic alterations in the generation of interictal epileptiform discharges (IEDs) (Köksal-Ersöz et al., 2022), fast ripples (Demont-Guignard et al., 2012), low-voltage fast onset (Molaee-Ardekani et al., 2010), and epileptic seizures (Kuchenbuch et al., 2021; Lopez-Sola et al., 2022). For example, physiologically based models have identified activity-dependent depolarizing GABA for the interictal-ictal transition, the role of the parvalbumin (PV) neuron in fast onset, and the loss of dendritic inhibition in the epileptogenic zone (Kurbatova et al., 2016).

To date, the majority of the proposed models have significant shortcomings in simulating realistic electrophysiological epileptic activity and often show low fidelity to clinical data. These models are often phenomenological descriptions lacking a substantial neurophysiological basis.

The fidelity of the epileptic signal simulation is important because the morphology of the recorded physiological or pathological EEG activity underlies mechanistic insights about the synaptic dynamics (Chizhov et al., 2019; Köksal-Ersöz et al., 2022). Unfortunately, the standard clinical scalp EEG uses a limited number of electrodes, which hinders the ability to accurately localize the sources of epileptic activity within the cortex. In addition, the personalization of the epileptogenic networks may be useful not only for understanding the neurophysiology underlying epileptic events on scalp EEG but also as a tool for planning and optimizing epilepsy therapy (Dallmer-Zerbe, Jiruska, et al., 2023).

We have recently developed a whole-brain computational model, named COALIA, based on the neurophysiology of different conscious states (Bensaid et al., 2019). The efficacy of COALIA was demonstrated by Bensaid et al. (2019), who compared the simulated cortical-level activity generated by the model with real EEG recordings obtained from awake and sleeping humans. The results showed that the simulated EEG mirrored closely the real EEG in terms of morphology, spectral content, and topographical voltage distribution. Overall, the use of COALIA provides a promising avenue for accurately localizing epileptogenic networks, which can ultimately aid in the diagnosis and treatment of epilepsy. However, accurately simulating patient-specific scalp EEG that respects the topology, the polarity, and the diffusion of IEDs is very challenging. The main limitations appear especially when it comes to integrating neuronal connectivity, cortical dynamics, and the patient’s anatomy, which will be projected onto virtual electrodes. To overcome these limitations, in this study, we use a novel version of COALIA. It integrates the recently developed neuronal mass model (NMM) (Köksal-Ersöz et al., 2022) that is adapted to the layered structure of the neocortex and to the patient’s anatomical head model contracted from neuroimaging. This digital brain can generate realistic scalp EEG specific for epileptic patients. To simulate the epileptic activity induced by the focal cortical dysplasia (FCD), we reproduced intracranial IEDs recorded with electrocorticography (ECoG) strips with a single layered NMM. Afterward, we tailored the extent of the epileptic zone (EZ) to simulate patient-specific scalp EEG with optimized similarity to real recordings, both before and after surgery.

Overall, we developed a fully personalized human brain model able to simulate a realistic scalp EEG and provide predictions regarding the neuronal mechanisms and the identification of the EZ.

## METHODS

### Clinical Data and Surgical Treatment

For this study, a 44-year-old patient with drug-resistant left focal frontal epilepsy was selected. We decided to select this case because she had a structural epilepsy related to a type 2 FCD (FCD 2), a pathology known for having a very high concordance between the irritative zone and the EZ (Chassoux et al., 2000). Moreover, the patient had a previous surgery, making her anatomy highly different from a standard atlas. The patient provided written informed consent for all procedures, analysis, and publication of anonymized clinical data in accordance with the Declaration of Helsinki. All proposed paraclinical investigations (neuroimaging, noninvasive and invasive EEG recordings) were in accordance with the best standard of care and not modified for research purposes.

The patient did not have any family history or personal risk factors for epilepsy during her development, such as febrile convulsions or head trauma. Her birth as well as her development were considered within normal limits. Seizures began at the age of 5 years, mostly as short, focal seizures with asymmetric tonic posturing during sleep. The patient underwent presurgical workup in her original country, including neuroimaging and video EEG monitoring that recorded several seizures. The patient underwent a workup that identified a left frontal structural epilepsy caused by an FCD 2 visible on MRI. At the age of 31 years, the patient was treated with an epilepsy surgery, which did not have an impact on seizures (Engel 4 B) as the resection was too anterior. At the age of 39 years, we started a second presurgical evaluation, which opted for a more posterior second intervention guided by intraoperative mapping (ECoG strips). The patient was completely seizure free at 2 years as well as at the last follow-up (Engel 1A), confirming that the EZ was successfully removed. The histopathological analysis revealed an aspect diagnostic for a FCD 2a on the resected tissue specimens (Najm et al., 2022). The patient had a moderate speech initiation difficulty and an expressive aphasia, probably because of the interception of the aslant tract (Catani et al., 2012). This deficit significantly improved after 6 months of speech therapy.

**Figure 1.**
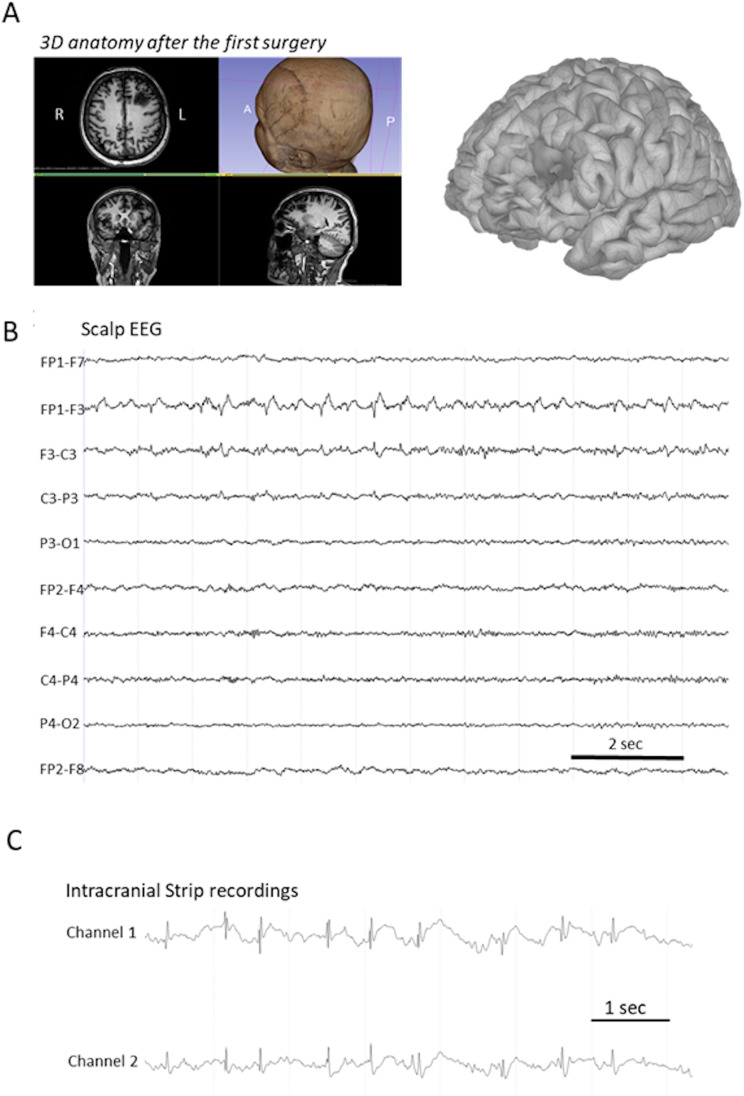
Presurgical investigations. (A) Illustration of the MRI and the 3D reconstruction of the cortex after the first surgery. (B) Illustration of the patient’s scalp EEG recording revealing prominent interictal spikes within the left frontal cortex, especially on FP1-F3 and F3-C3 electrode derivations. (C) An example of an ECoG recording showing interictal SW discharges on the borders of the supposed lesion on MRI.

Figure 1A depicts the T1-weighted MRI after the first unsuccessful surgery. The first surgery failed to remove the FCD in the dorsolateral prefrontal cortex located in the middle frontal gyrus. The interictal scalp EEG was registered with a modified 10 × 10 montage containing 32 electrodes, organized in a bipolar longitudinal montage, with a 512-Hz sampling rate. In accordance with the presumed lesion, the interictal scalp EEG showed continuous left parasagittal frontal spike-wave complexes with a phase reversal on the F3 electrode (Figure 1B). To acquire the direct intracranial recordings, 2 × 4 contact strips (Channels 1 and 2 in Figure 1C) were placed on the dorsal and ventral border of the suspected lesion (superior and middle frontal gyrus, behind the preexistent surgical scar). They were connected to a BRAIN QUICK portable EEG system. These recordings were performed under light anesthesia, in a bipolar manner, with a 1,024-Hz sampling rate, using the scalp as the reference. They confirmed the electrophysiological signature of the underlying dysplasia with a continuous 2- to 3-Hz spike and polyspike-wave complexes pattern. The regions displaying this pattern were completely removed with a final verification with the same pair of strips, confirming that the margins of the resection presented only slow elements without epileptiform anomalies or pathological fast rhythms. Based on current epilepsy surgery practices, the EZ was defined as being concordant with the part of the irritative zone needed to be removed for the complete disappearance of IEDs (Di Giacomo et al., 2019).

### Model

#### Neuronal mass model.

The local (mesoscale) dynamics of an “isolated” brain region are modeled by a neural mass formulation (Freeman, 1987; Jansen & Rit, 1995; Wendling et al., 2002). We have chosen this computational modeling, as even if it encounters limitations (Deschle et al., 2020), it allows a detailed analysis of the cortical physiology and can support hypothesis testing derived from empirical data (Bullock et al., 1977; Moran et al., 2013). This can be especially meaningful for epileptic pathophysiology, like the generations of interictal discharges (Wendling et al., 2016). The NMM assumes that dynamics of excitatory and inhibitory neuronal subpopulation can be described by their mean firing rate (Abbott & Chance, 2005). The so-called *transfer function* relates the incoming synaptic potentials *v* to the mean firing rate (output) and is a sigmoidal function accounting for the nonlinearities in neural dynamics, that is,

$$S\left(v\right)=2e_{0}/\left(1+e^{r}\left(v_{0}-v\right)\right);$$

where 2*e*_0_ is the maximum firing rate, *r* is the stiffness, and *v*_0_ is the potential at *e*_0_.

Synaptic interactions between the subpopulations are approximated by alpha functions that relate the average firing rates to average postsynaptic potentials (PSPs) given by the solution of the following differential equation:

$$\ddot{y}=W/\tau_{w}S\left(v\right)-2/\tau_{w}\dot{y}-1/\tau_{W}^{2}y;$$

where *W* is the average synaptic gain and *τ*_*w*_ reflects the kinetics of excitatory and inhibitory PSPs (EPSPs and IPSPs).

**Figure 2.**
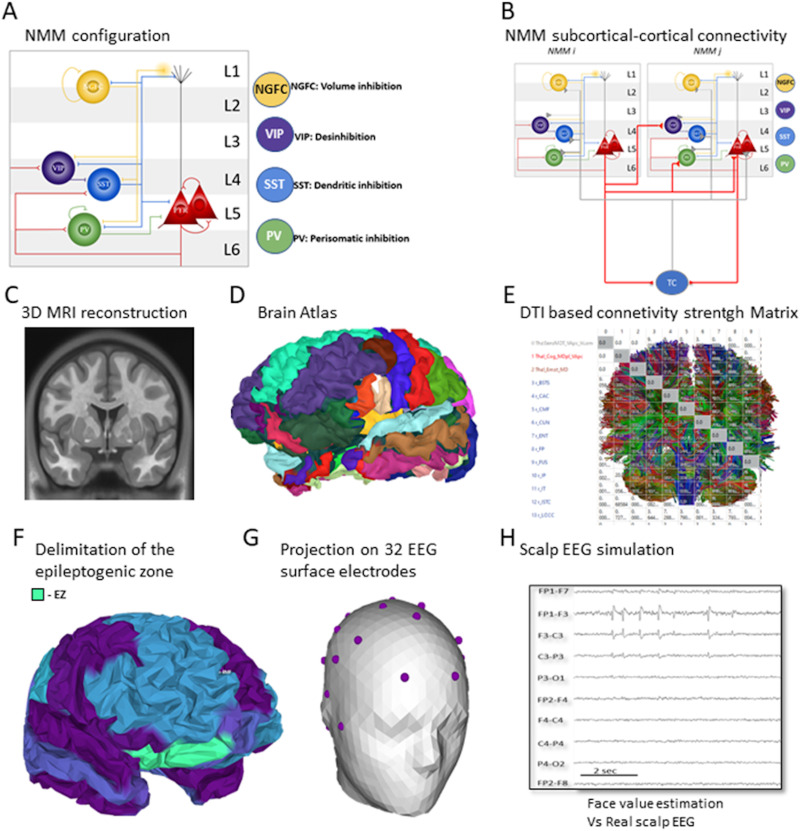
Epileptic COALIA (eCOALIA). (A) The neural mass model of a cortical column comprises two subpopulations of pyramidal neurons and four main types of GABAergic interneurons (PV, SST, VIP, and NGFC). This NMM is considered for modeling each cortical area of the atlas. (B) The connectivity between the cortical NMMs and subcortical regions, such as the thalamus, is established via long-range excitatory glutamatergic connections that target all cell types. The thalamic input to a cortical column is received by PYR, PV+, SST+, VIP+, and NGFC subpopulations. The thalamus receives excitatory input from the cortex. (C) The patient’s MRI is used to construct the head model. (D) Desikan-Killiany brain atlas is used to parcel the cortical surface. (E) Cortical and subcortical regions are connected via a DTI-based structural connectivity matrix and a delay matrix representing the communication delay between them. (F) The epileptogenic zone, which undergoes interictal epileptiform discharges, is defined after solving the inverse problem applied to scalp EEG signals. This region is associated with a NMM, which models the observed epileptic activity recorded with an ECoG, while the rest of the cortical regions are set in the background state. The source activity is projected on surface EEG electrodes (G) and simulated EEG signals are computed (H).

The NMM considered for the neocortical regions (Figure 2A) consists of two subpopulations of glutamatergic pyramidal neuron (PYR and PYR′) and four types of GABAergic interneuron subpopulations (i.e., neuroglia form cells [NGFC], vasoactive intestinal peptide (VIP), PV, and somatostatin [SST]-positive [+] interneurons), which are widely present in the neocortex (Jiang et al., 2015; Tremblay et al., 2016; Wamsley & Fishell, 2017). In this model, the PYR′ population accounts for the collateral excitation among the PYR neurons. The PV+ and SST+ interneurons are locally excited by the PYR, and in turn, they mediate fast GABAergic and slow GABAergic IPSPs, respectively. Finally, the PV+ interneurons are inhibited by the SST+ interneurons. The local cellular circuit motif is based on Womelsdorf et al. (2014).

The connectivity among these neuronal subpopulations (including both feedback and feedforward connections) was based on data reported in the literature (Eyal et al., 2018; Markram et al., 2015) and on the comprehensive review of the interlaminar connections in the neocortex by Thomson and Bannister (2003). The model features a single population of SST+ interneurons. However, the kinetics of the PSP varies depending on the targeted cell region. Specifically, the apical dendritic kinetics are slower than the basal dendritic kinetics (Köksal-Ersöz et al., 2022; Wendling et al., 2024).

The local network structure and the kinetics of synapses are deducted from the physiological information (Jiang et al., 2015; Shamas et al., 2018; Tremblay et al., 2016). The time constant parameter *τ*_*w*_ reflects the kinetics (rise and decay times) of the glutamatergic and the GABAergic PSPs of cortical neurons. The kinetics of the modeled PSPs reproduce those of the PSPs recorded in animal experiments: PYR EPSP from Zaitsev et al. (2012); PV+ and SST+ IPSP from Bacci et al. (2003), Deleuze et al. (2019), and Seay et al. (2020); VIP+ IPSP from Karnani et al. (2016); and NGFC+ IPSP from Oláh et al. (2007), Povysheva et al. (2007), and Wozny and Williams (2011). Model equations of the neocortical NMMs are provided in Supporting Information Equations S1–S3, with parameters in Supporting Information Tables S1–S2.

The layered structure of the neocortex is represented in the NMM (Köksal-Ersöz et al., 2022; Lopez-Sola et al., 2022; Sanchez-Todo et al., 2023), where the six layers of a human cortical column having a physiological thickness are considered. It is assumed that only the synaptic inputs on the layer V pyramidal neurons contribute to the current dipole generated by point contacts of the averaged synaptic locations. EEG signals originate from the cortical activity, typically from the synaptic inputs to pyramidal cells, which are geometrically aligned (Lopes da Silva, 2013). Briefly, synaptic inputs onto pyramidal cells cause change in extracellular potential by moving extracellular ion currents from current sources (reflection of intracellular hyperpolarization) to sinks (reflection of intracellular depolarization). If the recording electrode is sufficiently far from the current sources and sinks, as in the case of EEG electrodes, then the extracellular potential can be estimated by the *current dipole approximation* (Lindén et al., 2010; Næss et al., 2021; Nunez & Srinivasan, 2006):

$$\phi \left(r\right)=\frac{1}{4\sigma \pi }\frac{\sum_{k=1}^{M}I_{k}\left(t\right)\vec{d_{k}}\cos\left(\theta \right)}{R^{2}},$$

where *I*_*k*_(*t*) = *ηy*_*k*_(*t*) is the axial current, $\vec{d_{k}}$ is the distance vector traveled by the axial current, and cos(*θ*) is the angle between the current dipole and the distance vector from the source to the electrode. The parameter *σ* is the conductivity of the extracellular milieu, and *R* = ∣*r* − *r*_*source*_∣ is the distance between the source and the electrode. The parameter *η* = 10^−3^*S* is a conversion factor relating the PSP to the postsynaptic current (Lopez-Sola et al., 2022).

The NMM of the thalamus considers a simpler structure with a glutamatergic neuronal subpopulation—thalamocortical neurons (TC cells) and two types (slow and fast) of GABAergic neuronal subpopulations (reticular nuclei (RN) cells 1 and 2) (Mina et al., 2013) (Figure 2B). The model equations of the thalamus are given in Supporting Information Equations S4–S5, with parameters in Supporting Information Table S3.

#### Description of the cortical NMM and the whole-brain model.

Each microcircuit represented by a cortical NMM can generate distinct brain oscillations, such as alpha rhythms through the PYR-SST loop, gamma rhythms through the PYR-PV loop, delta rhythms through increased thalamocortical connectivity, disinhibition through VIP-SST microcircuits, and volume inhibition mediated by the NGFC. Neural masses are synaptically connected through long-range glutamatergic projections among pyramidal neurons and GABAergic interneurons (Bensaid et al., 2019).

The main connections between the thalamus and neocortex were taken into consideration in the model. As in classical thalamocortical models, the thalamic neural subpopulations (TC, RN_1_, RN_2_) receive glutamatergic inputs from PYR. In turn, PYR receive excitatory inputs from TCs, which is the only type of thalamocortical connection considered in the model. In terms of GABAergic cortical targets, thalamic projections target PV+ basket cells (Cruikshank et al., 2007; J.-M. Yang et al., 2013), SSTs (Ji et al., 2016; Tan & Bullock, 2008), and VIPs (Williams & Holtmaat, 2019; J.-J. Yang et al., 2017). It is noteworthy that time delays between the thalamus and the cortical NMMs were included as in the cortico-cortical long-range connections. The set of equations modeling the thalamocortical connections is described in the Supporting Information section (Supporting Information Equations S4–S5).

To generate the parameters of the epileptic NMM, we optimized the parameters within a biological framework and adapted the global modifications related to epilepsy. We begin from a physiologic NMM and we modify it within tight boundaries. We first start by changing the parameters of the amplitude of the EPSPs and the IPSPs generated by the five categories of neurons. Once we obtain a partial resemblance with the signal, the fine-tuning of the NMM is obtained by modifying the intra-NMM connectivity. The results are verified with the cross-correlation index, which offers feedback that permits us to maximize the resemblance.

At the global level, the large-scale model is constructed based on number of regions of interest (*nROIs*) = 68 regions of interest from the standard anatomical parcellation of the Desikan-Killiany atlas (Desikan et al., 2006) (the right and the left insula were excluded, leaving 66 brain regions). To these, one final subcortical NMM was added that represents the thalamus for a total of 67 brain regions (Figure 2D). In this case, each neural mass represents the local field potential (LFP) of one atlas region, in which the activity is assumed to be homogeneous. To generate the interictal scalp EEG simulation, the epileptic NMM, obtained previously, was introduced consecutively into the location of all cortical regions compatible with the scalp lateralization and location, and its connectivity was allocated. The scalp interictal epileptic spike (EEG-IES) similarity index, described in greater detail in the Similarity Index section, was employed to achieve the greatest possible resemblance to the patient’s interictal EEG. Once we obtained a partial resemblance with the signal, the fine-tuning is obtained by fractioning this area to best fit the FCD’s restricted volume.

#### Head model and cortical morphology.

The Boundary Element Method is employed to perform calculations within a realistic head model that incorporates the conductivity properties and geometry of the brain, the skull, and the scalp. This crucial step results in a 32 × 15,000 leadfield matrix (denoted as A), signifying the individual contribution of each of the 15,000 cortical dipoles to each of the 32 scalp electrodes. Subsequently, within this matrix, leadfield vectors pertaining to a shared region of the Desikan Atlas are aggregated, yielding a streamlined 66 × 32 matrix denoted as G. Readers can refer to Bensaid et al. (2019) for a more detailed description of the whole-brain model, so-called COALIA.

The cortical mesh was built in Brainstorm (Tadel et al., 2011) following a similar procedure with the one detailed by Bensaid et al. (2019). In summary, the ICBM152 2023b served as the template brain MRI scan, while patient-specific meshes were derived from the T1 MRI after the initial and subsequent surgeries, employing the FreeSurfer image analysis suite (Dale et al., 1999; Fischl et al., 1999) for a comprehensive 3D reconstruction. The brain mesh is used to distribute all the neural masses spatially over the cortex.

#### Connectivity.

In order to improve the realistic aspect of the simulated functional connectivity, COALIA was upgraded using the structural connectivity matrices averaged among a large number of healthy participants (487 adult subjects) through diffusor tensor imaging (DTI) as provided in the Human Connectome Project (https://www.humanconnectome.org/) (Van Essen et al., 2013). Hence, we defined our large-scale structural connectivity matrix that represents the density of fibers between all pairs of 66 cortical regions. Please note that when fragmenting a larger atlas region (as we did for the left frontal rostral middle gyrus (F-RMG) L-RMF, in order to best approximate the EZ), each subdivision guarded the same connectivity as the original area. We used the averaged fractional anisotropy measures to connect all the NMMs. The time delay was defined in the form of a matrix where the elements represent the Cartesian distance between cortical NMMs divided by the mean velocity of propagation for action potentials. Here, we used a mean velocity of 7.5 m/s. Connections related to the previously operated cortical area were annulled in the connectivity matrix.

A schematic overview of the upgraded COALIA model is presented in Figure 2. The eCOALIA software was made using Python3, and a graphical user interface was made with the PyQt6 module. The MRI of the patient was postprocessed with the Brainstorm toolbox (Tadel et al., 2011) used with MATLAB (The MathWorks, USA, version 2018b). It allows to compute the different 3D meshes and the parcellation (Desikan-Killiany) used in our model. The leadfield matrix used for the forward problem was computed with OpenMEEG (Gramfort et al., 2010). A minimal version of the eCOALIA software is available at https://github.com/ymmx2/eCOALIA. It is worthy to note that, in this study, we aim to use a previously validated model structure (COALIA; Bensaid et al., 2019) for testing methods included in the functional connectivity states estimation; however, this is the first study trying to simulate the pathophysiology related to an epileptic brain.

### Similarity Index

One method commonly used in the literature to quantify the similarity between two signals is the cross-correlation, which is defined as:

$$R\left(l\right)=\sum_{n=0}^{N}f\left(n\right)g\left(n+l\right);$$

where *l* is the lag between both signals. For this study, we chose an evaluation of the similarity of the simulated and recorded intracranial signals based on this function. We chose to attribute to our similarity index the maximum value of *R*, which allows a good correlation when events (like epileptic spikes) are not concomitant in both signals. This index takes values between −1 and 1. A value of 1 means that the similarity between the two signals is maximal (identical signals).

However, even if the cross-correlation index can be easily used on a single channel presenting spikes, it is uninformative for the similarity of the entire scalp EEGs that were generated for this study. For this purpose, we have employed a custom approach that quantifies the presence of spikes as well as their frequency and spatial distribution.

Initially, all IESs were identified on all channel derivations using an EEG window of 40 s of both real and simulated EEG data. For each derivation, IESs counting and the automatic measurement of their amplitude (the minimum and the maximum) were performed using an in-house Python script. After this quantification, the polarity of the spike and wave (SW) were labeled on each channel derivation for a bipolar longitudinal montage (i.e., positive spike-positive wave, positive spike-negative wave, negative spike-positive wave, and finally, negative spike-negative wave).

For a formal comparison, we developed a total score comprising seven criteria based on clinical neurophysiology practice, which we opt to call the EEG-IES similarity index. For a complete view of the scoring system please refer to Table 1.

**Table 1.** EEG-IES similarity index scoring for quantifying the resemblance of simulated 10–20 scalp EEGs presented in a bipolar longitudinal montage with the real EEG containing IESs

EEG-IES similarity index—Question	YES	NO	Reference
1) The channel derivation for which IES have the highest amplitude			
Are channels on real vs. simulated EEG identical?	1p	0p	/EEG
2) IES amplitude gradient			
Does the IESs’ amplitude evolution follow the same anteroposterior gradient?	5p	0p	/EEG
3) IES frequency			
Is the frequency of the IESs on the simulated EEG within the range of the measured frequency on real EEG (< ±50%)?	1p	0p	/channel
4) IES polarity			
For each channel in the simulated EEG, is the polarity (NP, PP, PN, NN) the same to the one observed in the real EEG?	1p	0p	/channel
5) IES presence			
Are the channels where the IESs appear on the simulated EEG identical to the real EEG?	1p	0p	/channel
6) IES absence			
Are the channels where the IESs are absent on the simulated EEG identical to the real EEG?	1p	0p	/channel
7) Phase inversion			
Is the common electrode, where the most prominent phase inversion (PN > NP) is found, identical?	5p	0p	/EEG

PP - positive spike-positive wave, PN - positive spike-negative wave, NP - negative spike-positive wave, and finally, NN - negative spike-negative wave.

Finally, the points were aggregated, resulting in a personalized score for each modeled EEG and a total theoretical number of points for each real EEG. The score was subsequently normalized and multiplied by 100, enabling its expression as a percentage of similarity between 0% and 100%.

## RESULTS

### The Simulation of the Intracranial Recordings

**Figure 3.**
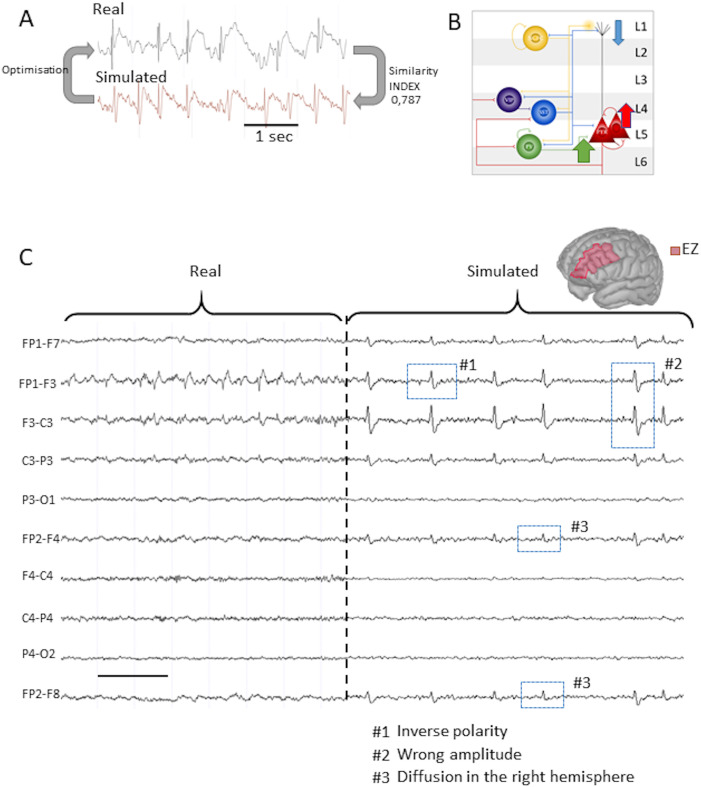
Simulated intracranial and scalp EEG. (A) Real intracranial ECoG recording versus simulated LFP. The signal was simulated in one population of NMM. After optimization, we found a similarity index of 0.787 between two random spike-wave discharges. The schema represents the variation of coupling coefficient compared with the generic parameters. (B) Layered NMM with synaptic modifications to render the model similar to the real FCD’s activity. (C) Real scalp EEG of the patient versus simulated EEG with a longitudinal montage. The signal was simulated with the COALIA model, which contains 66 interconnected populations of NMM, based on the Desikan Atlas. The interictal activity parameters were inserted in the NMM of the entire left F-RMG. The EEG-IES index is a low-to-moderate 43% score.

ECoG recordings were used to simulate the IEDs generated in the EZ. The tuning of the neural mass model (NMM)’s parameters was based on a similarity index based on a cross-correlation function computed on real and simulated SW recorded in EZ (Figure 3A) as described in the Similarity Index section. The results showed that the model could accurately reproduce SWs recorded in the EZ close to the FCD with an index of 0.787. The realistic SWs, such as those generated in the dysplasia, could be obtained by increasing the amplitude of the glutamatergic excitation, and the PV-mediated somatic inhibition, while at the same time decreasing the amplitude of the SST-mediated dendritic inhibition (Figure 3B and Supporting Information Table S1). Overall, to accurately reproduce the SW’s morphology, the excitation to dendritic inhibition ratio was greatly enhanced with respect to the background activity. To understand how this objective index performs in relation to subjective appreciation of the intracranial recordings, we generated three progressively less-similar ECoG recordings based on the same NMM configured to match our patient’s dysplasia. The results are presented in Supporting Information Figure S1.

### Template Brain With the EZ

For this study, the position of the ECoG strips recording the SW discharges was not known when the computational simulations were performed. However, since IEDs were clearly visible on the F3 scalp-EEG’s electrode, we decided to place the epileptic neural mass in different regions of the Desikan-Killiany atlas located in the left frontal cortex. The location of the epileptogenic NMM was determined to be approximately within the left frontal rostral middle gyrus (F-RMG). However, it was observed that if the EZ extended across the entire surface of the left F-RMG on the template brain’s anatomy, a comparison with actual EEG data that revealed that simulated IEDs exhibited an inverse polarity, inappropriate amplitude, and diffuse propagation toward the right hemisphere, projecting onto FP2-F8 electrodes (Figure 3C). Consequently, the EEG-IES index was relatively low at 43%.

**Figure 4.**
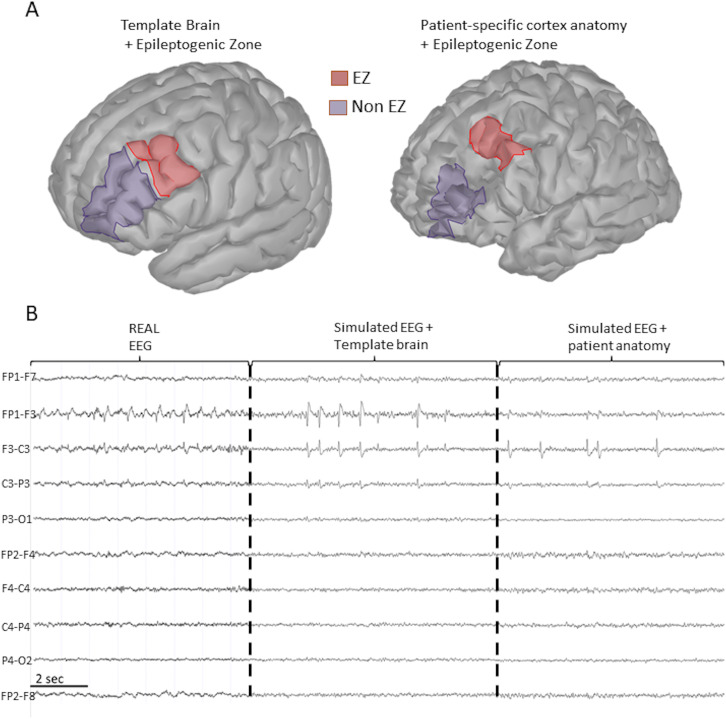
Anatomical model and scalp EEG variability. (A) Representation of a template cortex and the patient-specific cortex. The patient’s cortex was reconstructed from her MRI. The epileptogenic zone is represented in red. This region corresponds to a manual segmentation of the left rostral middle frontal gyrus. (B) Real scalp EEG of the patient versus simulated EEG with template cortex or with patient-specific cortex with longitudinal montage. In the simulations, the interictal activity was generated in the epileptogenic zone. The EEG-IES score was 92% for the atlas generated EEG and a 73% for the EEG generated with the patient’s mesh.

As a second step, we subdivided the scout representing the F-RMG of the template brain into two zones: the EZ with a NMM generating large epileptic spike-wave discharges (red zone; Figure 4A) and a non-EZ with a NMM generating background activity (purple zone; Figure 4A). When we limited the EZ to the posterior third of the left F-RMG, the simulated EEG highly resembles the real EEG (Figure 4B), reducing the propagation, reducing the amplitude of the IEDs, and respecting the global topography of the EEG. This improved in a significant manner the similarity with the real EEG, as also demonstrated by the quantified by the EEG-IES similarity index of 92%.

### Patient-Specific MRI and Simulation Optimization

This predicted EZ surface was then applied to the patient-specific MRI. The simulation results suggest that the cortex located behind the resected area from the previous surgery (Figure 4A, right panel) was responsible for the remaining epileptic activity recorded on the scalp EEG (Figure 4B) with a very high performance of similarity index of 92%. When the EZ (in red, Figure 4A) was delineated to the posterior part of the left F-RMG behind the resected tissue, in the patient-specific cortical mesh, with the optimal spatial extent, we succeeded in reproducing the principal characteristics of the scalp EEG. The topography of IED on electrodes FP1-F7, FP1-F3, F3-C3, and C3-P3 is highly similar to the real scalp EEG recordings (Figure 4B). This translated in a good EEG-IES index of 73%. Please note that this simulation underperformed the atlas-generated one. This is because, for the moment, the COALIA model does not include the electrical propagation through liquids, which is necessary when dealing with postsurgery brain breaches like for our patient.

### Real Versus Simulated Surgery

**Figure 5.**
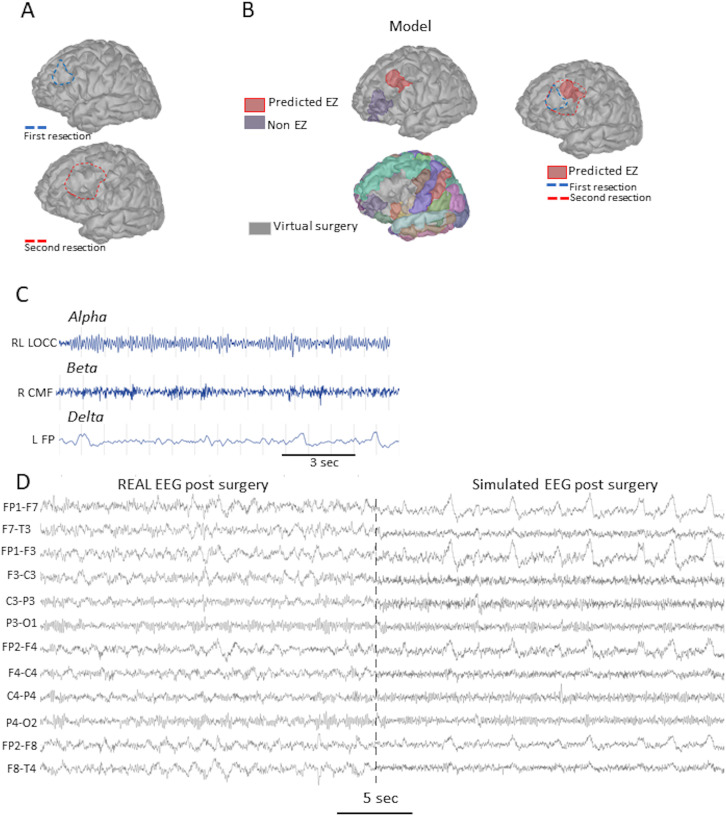
Simulation of scalp EEG after the second surgery. (A) Brain anatomy of the patient showing the first unsuccessful (blue line) and the second (red line) successful resection of the epileptogenic zone. (B) Illustration of the location (top, red area) of the epileptogenic zone according to our model simulations based on interictal activity. A simulated surgery was made by removing the epileptic NMM from the leadfield calculation (bottom). On the brain model situated in the right, we depicted the superposition between the real surgery and our predicted epileptogenic zone. (C) The add-on of the alpha, beta, and delta rhythms in the digital brain to improve the realistic appearance of the resting-state scalp EEG. (D) Comparison between the resting-state scalp EEG (eyes closed) recorded after the second surgery of the patient and the simulated scalp EEG after the in silico surgery (right panel).

The patient was operated a second time in the neurosurgery department of the Rennes University Hospital. The prediction of the model in terms of the EZ’s localization and spatial extension was confirmed by the results of the second surgery (Figure 5A). As shown in Figure 5B (middle and right panel), the resected region matches the EZ predicted by the model. Finally, if the operated cortical region was removed from the simulations, all the interictal spikes disappeared from the simulation (Figure 5D). The real EEG postoperative recording of the patient was performed with the eyes closed, generating prominent alpha oscillations. To improve the concordance with the resting-state EEG, alpha, beta, and delta rhythms were added into the model (Figure 5C). These were obtained by several distinct manipulations. The alpha rhythm was obtained by increasing the SST-mediated dendritic inhibition in the occipital visual areas (right and left occipital pole, inferotemporal, and the lingual gyrus). The beta rhythm was obtained by increasing the somatic inhibition in the frontal cortex (rostral middle gyrus, caudal middle gyrus). The delta rhythm was obtained by decreasing the time constant of the PSPs in the left frontal pole, as the opted NMM lumps the axonal delays, which is proportional to the size of interacting regions, which is large in the case of the delta band rhythms. Related model parameters are given in Supporting Information Table S2. Overall, if the same resection is simulated on the model, the real postoperative scalp EEG and the virtual one were very similar as evaluated by the authors certified in clinical neurophysiology. However, this cannot be objectively quantified with the same metrics, as the EEG-IES index becomes 0 when the recording does not contain any IES. An objective comparison metric of evaluating “near-normal/not-epileptic scalp EEG” is beyond the scope of this study.

## DISCUSSION

We have recently developed a neurophysiologically based whole-brain digital model capable of generating realistic scalp EEG (Bensaid et al., 2019; Tabbal et al., 2022). In this study, a whole-brain model integrating a novel layered neocortical NMM was able to reproduce real intracranial recordings from a patient diagnosed with FCD, with a high degree of similarity. The epileptic cortical area was precisely positioned and expanded to simulate the epileptogenic zone, which induced IEDs in the model. Subsequently, by projecting the interictal epileptic activity onto the simulated scalp EEG, different topographical patterns of the scalp EEG were observed. By aligning the topography of the simulated EEG with the actual scalp EEG recordings, the virtual model served as a tool for localizing the EZ and determining its extent. The match between the resected area during the second surgery and the predicted epileptogenic zone validated the model’s predictions. It is important to acknowledge that this surgery offered seizure freedom to the patient.

In this study, we predicted the localization and the extent of the epileptogenic zone based on the shape, polarity, amplitude, and diffusion of the IEDs projected on the scalp using the forward problem. To successfully match the topography of the real interictal clinical EEG recording, different scenarios were used in the model. Despite the large number of possible parameter combinations, only a few solutions emerged. Choosing a large surface area of the EZ resulted in abnormally high-amplitude IEDs or in mismatched propagation of the IEDs to distant electrodes. Placing the EZ in other nearby cortical areas produced IEDs with opposite polarity or inconsistent localizations. Thus, the constraints effectively reduced the number of configurations that accurately replicated the patient’s interictal EEG recording.

However, as a general rule, IEDs diffuse outside the epileptogenic zone, and IEDs are usually not good biomarkers to identify the seizure onset zone (Bartolomei et al., 2016). In the selected clinical case, we deliberately chose an FCD 2 pathology as this model presents an advantageous scenario for simulating a constrained epileptogenic network (Di Giacomo et al., 2019). Even if it this article is a single-case presentation, we consider it more of a proof-of-concept work, as all FCD 2 have similar interictal behavior (Chassoux et al., 2000). This type of lesion can be easily modeled following the same pipeline. Moreover, it represents one of the leading causes of drug-resistant epilepsy necessitating surgical treatment. A comprehensive review article estimates it as representing 29%–39% of all FCDs, which in itself is the leading cause of drug-resistant epilepsy in children and the second leading cause in the adult population (Sisodiya et al., 2009). We propose that it can be especially useful for MRI-negative FCD 2. Using IEDs to delineate the epileptogenic zone cannot be generalized to other forms of epilepsies (Kobulashvili et al., 2018).

To mimic the shape of the IEDs produced by a FCD, the NMM’s parameters were tuned with an exceptionally high excitation-inhibition ratio, related to very high glutamatergic and very low dendritic targeting of the SST-GABAergic synaptic strength. This result is in accordance with several studies showing that the FCD 2 presents an alteration of the GABAergic receptors’ expression (Crino et al., 2001), a depolarizing GABA current (Shao et al., 2022), a dysfunction of GABAergic synaptic inhibition (Calcagnotto et al., 2005), and a high level of an extrasynaptic NMDA-R subtype NR2B (Finardi et al., 2006; Ying et al., 2004). Profound alterations occur in both neuronal morphology and synaptic networks at the core of FCD 2, with a particular emphasis on the type 2b, leading to significant neuronal rearrangements and hyperexcitability (Rossini et al., 2021).

Considering personalized brain modeling in epilepsy, even though the field is relatively young, several models have been developed to help clinicians for better decision-making in epilepsy surgery (Jirsa et al., 2014; Junges et al., 2020; Kini et al., 2019; Nissen et al., 2021). Most of them integrate clinical data such as anatomy extracted from the T1 MRI, structural connectivity extracted from the DTI, and electrophysiological features like the organization of the epileptic network or ictal EEG derived from intracranial studies (stereo-electroencephalography (SEEG), ECoG). Some give the possibility to perform virtual resections with a significant predictive value of postsurgical results (Makhalova et al., 2022). Furthermore, other studies provide arguments for a genuine possible amelioration of the nowadays “state-of-the-art” resection proposal in the tertiary epilepsy centers: a reduction of the cortical volume of the initial resection proposal, sparing of nonnecessary fiber tracts, and even discovering epileptogenicity in nonimplanted additional areas in case of a failed first surgery (Kini et al., 2019; Li et al., 2018; Millán et al., 2022; Proix et al., 2017; Wang et al., 2023). We highlight several differences between these approaches and our approach. The first one concerns the computational models used to simulate the source activity. The mathematical models used in these simulations are phenomenological models that simulate various types of seizure-like patterns (Saggio et al., 2020) or seizure propagation behavior like in the epidemiological model (Millán et al., 2022, 2023). These models do not give access to neurophysiological insights as the neuronal types, and so far, it has not been shown that they can simulate interictal EEG activity by respecting the IEDs’ morphology. The cortical NMM proposed in this study is a neurophysiologically plausible model that integrates different cell types, their activities, as well as the most important synaptic interactions. Thanks to the biophysical computation of the LFP, the cortical NMM can simulate not only ictal activity (Lopez-Sola et al., 2022) but also IEDs with fine precision (Köksal-Ersöz et al., 2022, 2024). Moreover, the capacity of the model to simulate a wide range of cortical oscillations offers a unique opportunity for studying different cortical dynamics in epileptic patients. Another difference is that most studies proposing a patient-specific modeling are based on intracranial seizure recordings, whereas our approach is not restricted to intracranial recordings or seizure patterns, but it can consider scalp and invasive recordings relying also on interictal activity. A digital brain developed from the scalp interictal activity is considered to have considerable flexibility for clinical use as most patients do not benefit from intracranial recordings (Lang et al., 2024).

### Limitations and Further Research

In our study, the model’s parameters and patches simulating interictal events were optimized manually, which is time consuming. This limitation could be overcome by deploying an automatic model fitting pipeline, similar to the work of Dallmer-Zerbe, Jajcay, et al. (2023). Clinical research has been benefiting from artificial intelligence and computational modeling approaches (An et al., 2020). A generative computational model that simulates a wide range of IEDs can be used to generate large amounts of data for training feature classification algorithms to classify EZs and non-EZs and for improving EEG source imaging, as described in Sun et al. (2022). Patient-specific modeling can be used to predict surgical outcomes, functional alteration caused by medications, and the effects of therapeutic interventions, such as brain stimulation (Liu & Li, 2022; McIntyre & Foutz, 2013).

In this study, structural connectivity was based on a template derived from the Human Brain Connectome data imaging, but it could be tailored to individual patients by incorporating their own DTI data. Another interesting approach is to consider personalized matrices or atlases of effective connectivity or mixed structural-effective connectivity as it is known that not all the tracts extracted from the DTI are actually functioning connections (Donos et al., 2016; Lemaréchal et al., 2022; Shamir & Assaf, 2023).

Ultimately, the comparison between real and simulated data was computed in this study for a single channel and for a complete scalp EEG containing epileptiform spikes, focusing on replicating the morphology and the distribution of intracranial IEDs. In the future, to comprehensively assess the resemblance between real and simulated scalp EEGs in all conditions, across all derivations, the development of additional similarity indexes is warranted.

The study personalizes a previously validated, physiology-inspired brain model for consciousness states and epileptic events (both IEDs and seizures) by importing a real patient’s anatomy and intracranial EEG from the FCD tissue, recorded in the operatory room. This NMM could generate scalp EEGs with realistic accuracy and predicted the location of the lesion in the patient’s left frontal lobe. To quantify the resemblance, we developed two intuitive and normalized EEG similarity indexes: one for single-channel recordings useful for intracranial monitoring and the second for complete scalp EEG traces. These indexes can easily be employed by other modeling groups to evaluate their results in the future. Furthermore, the predicted location was validated with surgery, which resulted in seizure freedom. The parameters of the epileptic NMN reflected an exceptionally high excitation-inhibition ratio, related to very high glutamatergic and very low dendritic targeting of the SST-GABAergic synaptic strength. After removing the epileptic NMM in an in silico surgery, the simulated scalp EEG continued to resemble the real postsurgical clinical EEG and demonstrated the complete disappearance of the epileptiform activity. Our study, although for the moment limited to a specific pathology, offers a novel concept of neurophysiology-inspired brain modeling that could be integrated in the presurgical evaluation and postsurgical follow-up of drug-resistant epilepsy.

## ACKNOWLEDGMENTS

We would like to thank Mr. Arthur Bertin for his help and support in data simulations.

## SUPPORTING INFORMATION

Supporting information for this article is available at https://doi.org/10.1162/netn_a_00418.

## AUTHOR CONTRIBUTIONS

Mihai Dragos Maliia: Conceptualization; Data curation; Investigation; Methodology; Validation; Visualization; Writing – original draft. Elif Köksal-Ersöz: Conceptualization; Investigation; Methodology; Software; Visualization; Writing – original draft. Adrien Benard: Investigation; Methodology; Validation; Visualization; Writing – review & editing. Tristan Calas: Data curation; Visualization. Anca Nica: Validation; Visualization; Writing – review & editing. Yves Denoyer: Writing – review & editing. Maxime Yochum: Investigation; Methodology; Software; Writing – original draft. Fabrice Wendling: Project administration; Resources; Software; Writing – review & editing. Pascal Benquet: Conceptualization; Data curation; Formal analysis; Investigation; Methodology; Project administration; Visualization; Writing – original draft.

## FUNDING INFORMATION

HORIZON EUROPE European Research Council (https://dx.doi.org/10.13039/100019180), Award ID: 855109.

## DATA AND CODE AVAILABILITY

Imaging and electrophysiological data are clinical, so for public access, further HIPPA/institutional agreement is needed for raw images and recordings. A condensed version of the COALIA software is currently accessible at https://github.com/ymmx2/eCOALIA.

## Supplementary Material



## TECHNICAL TERMS

Interictal epileptiform discharges (IEDs): Anomalies in the EEG recording between the seizures, specific for patients with epilepsy like sharp waves, spikes, or polyspikes.
Epileptogenic zone: Zone considered essential for seizure generation and organization in focal epilepsy that needs to be surgically removed for curing the patient.
Neuronal mass model (NMM): Computer model at an intermediate scale that simulates the collective activity of large populations of neurons to study the brain.
Focal cortical dysplasia (FCD): Congenital malformation of brain development caused by a migration failure of neurons that is a frequent cause of drug-resistant epilepsy.
Electrocorticography (ECoG): Invasive neurophysiological method that records the brain extracellular activity with electrodes placed directly on the cerebral cortex.
Transfer function (for synapses): Describes the mathematical relationship between presynaptic input and postsynaptic output thus characterizing synaptic kinetics.
Excitatory postsynaptic potentials (EPSPs): A depolarizing change in membrane potential that increases the likelihood of cellular firing obtained by excitatory neurotransmitters.
Inhibitory postsynaptic potentials (IPSPs): A hyperpolarizing change in membrane potential that decreases the likelihood of cellular firing obtained by inhibitory neurotransmitters.
Cross-correlation function: Function that quantifies the degree of similarity between two signals as a function of their relative time lag.
