# Supplementary material for: Localization of the epileptogenic network from scalp EEG using a patient-specific whole-brain model
Source: Netw Neurosci. 2025 Mar 3;9(1):18–37. doi: 10.1162/netn_a_00418 (PMC11949544; doi:10.1162/netn_a_00418)
Supplement: Supplementary file 1 [file netn-9-1-18-s001.pdf]

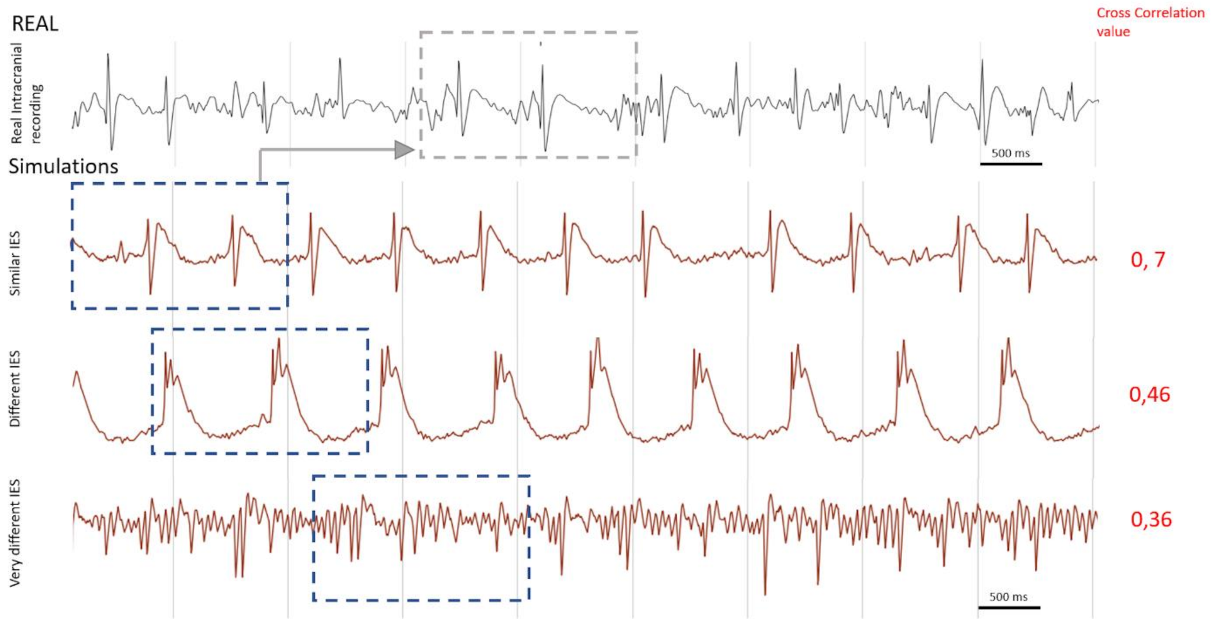

Supplementary figure: evaluation of the cross-correlation similarity index performance.

For this analysis, we took the NMM already configured to match the patient's dysplasia and we modified the connections to generate a lesser and lesser similar intracranial EEG according to subjective neurophysiological criteria.

The first simulation showed interictal spikes (positive spike followed by a positive wave) close to the real signal obtained by intracranial recording. The second configuration showed IES with a different shape. The third simulation is very different from real data showing high frequency oscillations with superimposed bursts of spikes.

We measured the cross-correlation values by comparing two consecutive spikes with a sliding window (of the time duration of one sampling size, which in this case was of 512 Hz) on the real

signal and obtain a score of maximal CC. The final value is the maximal value obtained for each of the three channels.

Values are 0.7 for the intracranial EEG containing similar spike and wave complexes, 0.46 for the one with moderately different spike and wave complexes, and 0.36 for the third one with different IES.

For our patient we obtained 0.787 value which was the maximal value that because of the noise in the signal.

Model equations

The system of equations of the laminar NMM representing a neocortical brain region is given by

$$\ddot{y}_{PYR} = \frac{W_{PYR}}{\tau_{PYR}} S\big(y_{PYR'} - C_{PV\to PYR} y_{PV} - C_{SST_B\to PYR} y_{SST_B} - C_{SST_A\to PYR} y_{SST_A} - C_{NGFC\to PYR} y_{NGFC} + y_{CC\to PYR} + y_{TC\to PYR}\big) - \frac{2}{\tau_{PYR}} \dot{y}_{PYR} - \frac{1}{\tau_{PYR}^2} y_{PYR}, \tag{S1a}$$

$$\ddot{y}_{PYR'} = \frac{W_{PYR}}{\tau_{PYT}} \left(p(t) + C_{PYR'\to PYR} S(C_{PYR\to PYR'} y_{PYR})\right) - \frac{2}{\tau_{PYR}} \dot{y}_{PYR'} - \frac{1}{\tau_{PYR}^2} y_{PYR'}, \tag{S1b}$$

$$\ddot{y}_{PV} = \frac{W_{PV}}{\tau_{PV}} S\big(C_{PYR\to PV} y_{PYR} - C_{PV\to PV} y_{PvV} - C_{SST_B\to PV} y_{SST_B} - C_{NGFC\to PV} y_{NGFC} + + y_{CC\to PV} + y_{TC\to PV}\big) - \frac{2}{\tau_{PV}} \dot{y}_{PV} - \frac{1}{\tau_{PV}^2} y_{PV}, \tag{S1c}$$

$$\ddot{y}_{SST_B} = \frac{W_{SST_B}}{\tau_{SST_B}} S(C_{PYR\to SST} y_{PYR} - C_{VIP\to SST} y_{VIP} - C_{NGFC\to SST} y_{NGFC} + y_{CC\to SST} + y_{TC\to SST}) - \frac{2}{\tau_{SST_B}} \dot{y}_{SST_B} - \frac{1}{\tau_{SST_B}^2} y_{SST_B}, \tag{S1e}$$

$$\ddot{y}_{SST_A} = \frac{W_{SST_A}}{\tau_{SST_A}} S(C_{PYR\to SST} y_{PYR} - C_{VIP\to SST} y_{VIP} - C_{NGFC\to SST} y_{NGFC} + y_{CC\to SST} + y_{TC\to SST}) - \frac{2}{\tau_{SST_A}} \dot{y}_{SST_A} - \frac{1}{\tau_{SST_A}^2} y_{SST_A}, \tag{S1f}$$

$$\ddot{y}_{VIP} = \frac{W_{VIP}}{\tau_{VIP}} S\big(C_{PYR\to VIP} y_{PYR} - C_{SST_B\to VIP} y_{SST_B} - C_{NGFC\to VIP} y_{NGFC} + y_{CC\to VIP} + y_{TC\to VIP}\big) - \frac{2}{\tau_{VIP}} \dot{y}_{VIP} - \frac{1}{\tau_{VIP}^2} y_{VIP}, \tag{S1g}$$

$$\ddot{y}_{NGFC} = \frac{W_{NGFC}}{\tau_{NGFC}} S\big(-C_{SST_B\to NGFC} y_{SST_B} - C_{NGFC\to NGFC} y_{NGFC} + y_{CC\to NGFC} + y_{TC\to NGFC}\big) - \frac{2}{\tau_{NGFC}} \dot{y}_{NGFC} - \frac{1}{\tau_{NGFC}^2} y_{NGFC}, \tag{S1h}$$

Function $S(v) = 2e_0/(1 + e^r(v_0 - v))$ corresponds to the wave-to-pulse function (see Methods 2.2.1) with parameters $(e_0, r, v_0) = (2.5\ s^{-1}, 0.56, 6\ V)$, which are kept at their standard values

(Freeman 1987; Jansen BH, Rit VG 1995; Wendling et al 2002). The same sigmoid funtions are considered for the thalamic population model below.

The unspecific excitatory cortical input $p(t) = p_m + \xi(t)$ follows a normal distribution with mean p_m and $\xi(t) = N(0, \sigma^2)$ and variance σ^2. Parameter values are given in *Table S1 and Table S2.*

The excitatory cortico-cortical inputs on subpopulations are given by

$$\ddot{y}_{CC\to PYR} = \frac{W_{PYR}}{\tau_{PYR}} \Sigma_{n=1,n\neq m}^{\mathrm{N}} C_{n\to m,PYR} F_n - \frac{2}{\tau_{PYR}} \dot{y}_{CC\to PYR} - \frac{1}{\tau_{PYR}^2} y_{CC\to PYR} \tag{S2a}$$

$$\ddot{y}_{CC\to PV} = \frac{W_{PYR}}{\tau_{PYR}} \Sigma_{n=1,n\neq m}^{\mathrm{N}} C_{n\to m,PV} F_n - \frac{2}{\tau_{PYR}} \dot{y}_{CC\to PV} - \frac{1}{\tau_{PYR}^2} y_{CC\to PV} \tag{S2b}$$

$$\ddot{y}_{CC\to SST} = \frac{W_{PYR}}{\tau_{PYR}} \Sigma_{n=1,n\neq m}^{\mathrm{N}} C_{n\to m,SST} F_n - \frac{2}{\tau_{PYR}} \dot{y}_{CC\to SST} - \frac{1}{\tau_{PYR}^2} y_{CC\to SST} \tag{S2c}$$

$$\ddot{y}_{CC\to VIP} = \frac{W_{PYR}}{\tau_{PYR}} \Sigma_{n=1,n\neq m}^{\mathrm{N}} C_{n\to m,VIP} F_n - \frac{2}{\tau_{PYR}} \dot{y}_{CC\to VIP} - \frac{1}{\tau_{PYR}^2} y_{CC\to VIP} \tag{S2d}$$

$$\ddot{y}_{CC\to NGFC} = \frac{W_{PYR}}{\tau_{PYR}} \Sigma_{n=1,n\neq m}^{\mathrm{N}} C_{n\to m,NGFC} F_n - \frac{2}{\tau_{PYR}} \dot{y}_{CC\to NGFC} - \frac{1}{\tau_{PYR}^2} y_{CC\to NGFC} \tag{S2e}$$

where F_n represents the firing rate of a presynaptic glutamatergic subpopulation of a neocortical region n and $C_{n\to m,i}$ is the coupling coefficient from the region n to the subpopulation i = {PYR, PV, SST, VIP, NGFC} of the region m as defined by the structural connectivity matrix.

Thalamo-cortical input is assumed to be only on the PYR subpopulation of a neocortical region and it is given by

$$\ddot{y}_{TC\to PYR} = \frac{W_{PYR}}{\tau_{PYR}} C_{TC\to m,PYR} F_{TC} - \frac{2}{\tau_{PYR}} \dot{y}_{TC\to PYR} - \frac{1}{\tau_{PYR}^2} y_{TC\to PYRC}\,, \tag{S3a}$$

$$\ddot{y}_{TC\rightarrow PV} = \frac{W_{PYR}}{\tau_{PYR}} C_{TC\rightarrow m,PV} F_{TC} - \frac{2}{\tau_{PYR}} \dot{y}_{TC\rightarrow PV} - \frac{1}{\tau_{PYR}^2} y_{TC\rightarrow PV}\,, \tag{S3b}$$

$$\ddot{y}_{TC\rightarrow SST} = \frac{W_{PYR}}{\tau_{PYR}} C_{TC\rightarrow m,SST} F_{TC} - \frac{2}{\tau_{PYR}} \dot{y}_{TC\rightarrow SST} - \frac{1}{\tau_{PYR}^2} y_{TC\rightarrow SST}\,, \tag{S3c}$$

$$\ddot{y}_{TC\rightarrow VIP} = \frac{W_{PYR}}{\tau_{PYR}} C_{TC\rightarrow m,VIP} F_{TC} - \frac{2}{\tau_{PYR}} \dot{y}_{TC\rightarrow VIP} - \frac{1}{\tau_{PYR}^2} y_{TC\rightarrow VIP}, \tag{S3d}$$

$$\ddot{y}_{TC\rightarrow NGFC} = \frac{W_{PYR}}{\tau_{PYR}} C_{TC\rightarrow m,NGFC} F_{TC} - \frac{2}{\tau_{PYR}} \dot{y}_{TC\rightarrow NGFC} - \frac{1}{\tau_{PYR}^2} y_{TC\rightarrow NGFC}\,, \tag{S3e}$$

Where F_{TC} represents the firing rate of the thalamic TC subpopulation in Eq (S4a) below. Parameter $C_{TC\rightarrow m,i}$ is the coupling coefficient from the TC subpopulation to the subpopulation i = {PYR, PV, SST, VIP, NGFC} of the region m as defined by the structural connectivity matrix.

System equations representing the thalamus are given by

$$\ddot{y}_{TC} = \frac{W_{TC}}{\tau_{TC}} S\left(y_{CT\rightarrow TC} + C_{TC\rightarrow TC} y_{TC} - C_{RT1\rightarrow TC} y_{RT1} - C_{RT2\rightarrow TC} y_{RT2} + p_{f,TC}\right) - \frac{2}{\tau_{TC}} \dot{y}_{TC} - \frac{1}{\tau_{TC}^2} y_{TC} \tag{S4a}$$

$$\ddot{y}_{RN1} = \frac{W_{RN1}}{\tau_{RN1}} S(y_{CT\rightarrow RN1} + C_{TC\rightarrow RN1} y_{TC}) - \frac{2}{\tau_{RN1}} \dot{y}_{RN1} - \frac{1}{\tau_{RN1}^2} y_{RN1} \tag{S4b}$$

$$\ddot{y}_{RN2} = \frac{W_{RN2}}{\tau_{RN2}} S(y_{CT\rightarrow RN2} + C_{TC\rightarrow RN2} y_{TC}) - \frac{2}{\tau_{RN2}} \dot{y}_{RN2} - \frac{1}{\tau_{RN2}^2} y_{RN2} \tag{S4c}$$

$$\ddot{p}_{f,TC} = \frac{W_{TC}}{\tau_{TC}} p_{TC}(t) - \frac{2}{\tau_{TC}} \dot{p}_{f,TC} - \frac{1}{\tau_{TC}^2} p_{f,TC} \tag{S4d}$$

The variable $p_{f,TC}$ is the thalamic noise. The cortico-thalamic inputs are given by

$$\ddot{y}_{CT\rightarrow TC} = \frac{W_{TC}}{\tau_{TC}} \Sigma_n^{\mathrm{N}} C_{n\rightarrow Th,TC} F_n - \frac{2}{\tau_{TC}} \dot{y}_{CT\rightarrow TC} - \frac{1}{\tau_{TC}^2} y_{CT\rightarrow TC} \tag{S5a}$$

$$\ddot{y}_{CT\rightarrow RN1} = \frac{W_{TC}}{\tau_{TC}} \Sigma_n^{\mathrm{N}} C_{n\rightarrow Th,RN1} F_n - \frac{2}{\tau_{TC}} \dot{y}_{CT\rightarrow RN1} - \frac{1}{\tau_{TC}^2} y_{CT\rightarrow RN1} \tag{S5b}$$

$$\ddot{y}_{CT\rightarrow RN2} = \frac{W_{TC}}{\tau_{TC}} \Sigma_n^{\mathrm{N}} C_{n\rightarrow Th,RN2,} F_n - \frac{2}{\tau_{TC}} \dot{y}_{CT\rightarrow RN2} - \frac{1}{\tau_{TC}^2} y_{CT\rightarrow RN2} \tag{S5c}$$

where F_n represents the firing rate of a presynaptic glutamatergic subpopulation in the neocortical region n, and $C_{n\rightarrow Th,i}$ is the connectivity from the region n to the thalamic subpopulations i={TC, RN1, RN2}. Parameter values are given in *Table S3.*

Table S1. Parameter set used to simulate Fig. 3, 4

Activity	Background activity	IED	Beta rhythm parameters
PSP amplitude (W_i)			
W_{PYR} (mV)	2.0	7.5	3.5
W_{SST_B} (mV)	30.0	25.0	13.0
W_{SST_A} (mV)	30.0	25.0	20.0
W_{PV} (mV)	30.0	40.0	18.0
W_{VIP}(mV)	5	5	5
W_{NGFC}(mV)	5	5	5
PSP time constant (τ_w)			
τ_{PYR} (s^{-1})	100.0	100.0	100.0
τ_{SST_B}(s^{-1})	30.0	30.0	30.0

τ_{SST_A} (s^{-1})	30.0	30.0	30.0
τ_{PV}(s^{-1})	150.0	150.0	150.0
τ_{VIP}(s^{-1})	20	20	20
τ_{NGFC}(s^{-1})	5	5	5
Coupling coefficient			
$C_{PYR \to PYR'}$	135.0	150.0	135.0
$C_{PYR' \to PYR}$	100.0	150.0	100.0
$C_{SSTA \to PYR}$	20.0	28.0	10.0
$C_{SSTB \to PYR}$	20.0	40.0	10.0
$C_{PV \to PYR}$	50.0	100.0	50.0
$C_{NGFC \to PYR}$	0	0	
$C_{PYR \to PV}$	50.0	50.0	60.0

$C_{PV\to PV}$	200.0	20.0	2.0
$C_{SSTB\to PV}$	13.5	25.0	7.0
$C_{NGFC\to PV}$	0	0	0
$C_{PYR\to SST}$	20.0	40.0	30.0
$C_{VIP\to SST}$	20	0	
$C_{NGFC\to SST}$	0	0	
$C_{PYR\to VIP}$	0	0	
$C_{SSTB\to VIP}$	20	0	
$C_{NGFC\to VIP}$	0	0	
$C_{SST\to NGFC}$	0	0	
$C_{NGFC\to NGFC}$	0	0	
External input $p(t)$			

mean (Hz)	120.0	128.0	120.0
Standard deviation (Hz)	2.0	4.0	6.0
Constants			
e_0	$2.5\ s^{-1}$		
r	0.56		
v_0	$6\ V$		

Table S2 Parameter set used to simulate Fig. 5

Activity	Alpha	Beta/Gamma	Local Delta
Region	RL IT, RL Locc, RL Ling, RL PeriCalc	R RMF, L CMF	L Frontopolar
PSP amplitude (W_i)			
W_{PYR} (mV)	4.5	5.3	0.88
W_{SST_B} (mV)	50	5	35
W_{SST_A} (mV)	4	5	5
W_{PV} (mV)	5	30	30
W_{VIP}(mV)	5	5	5
W_{NGFC}(mV)	5	5	5
PSP time constant (τ_w)			
τ_{PYR} (s^{-1})	100	100	15

τ_{SST_B}(s^{-1})	30	30	30.0
τ_{SST_A} (s^{-1})	30	30	5
τ_{PV}(s^{-1})	150	150	150.0
τ_{VIP}(s^{-1})	20	20	20
τ_{NGFC}(s^{-1})	5	5	5
Coupling coefficient			
$C_{PYR \to PYR'}$	200	60	20
$C_{PYR' \to PYR}$	100	150	10
$C_{SSTA \to PYR}$	20	20	14
$C_{SSTB \to PYR}$	20	20	2
$C_{PV \to PYR}$	50	25	200
$C_{NGFC \to PYR}$	0	0	0

$C_{PYR \to PV}$	50	150	50
$C_{PV \to PV}$	5	25	200
$C_{SSTB \to PV}$	60	13.5	25
$C_{NGFC \to PV}$	0	0	0
$C_{PYR \to SST}$	20	20	20
$C_{VIP \to SST}$	20	20	20
$C_{NGFC \to SST}$	0	0	0
$C_{PYR \to VIP}$	0	0	0
$C_{SSTB \to VIP}$	20	20	20
$C_{NGFC \to VIP}$	0	0	0
$C_{SST \to NGFC}$	0	0	0
$C_{NGFC \to NGFC}$	0	0	0

External input $p(t)$			
mean (Hz)	120	120	60
Standard deviation (Hz)	4	2	10

Table S3 Parameter of the thalamic population given in Eq (S4).

PSP amplitude	
W_{TC}(mV)	4.5
W_{RN1}(mV)	50
W_{RN2}(mV)	5
PSP time constant	
τ_{TC}(s^{-1})	100
τ_{RN1} (s^{-1})	30
τ_{RN2} (s^{-1})	150
Coupling coefficient	
$C_{TC \to TC}$	200
$C_{RN1 \to TC}$	20
$C_{RN2 \to TC}$	20

$C_{TC \to RN1}$	20
$C_{TC \to RN2}$	60
External input $p_{TC}(t)$	
mean (Hz)	120
Standard deviation (Hz)	4
